# Correction: Transgenic shRNA pigs reduce susceptibility to foot and mouth disease virus infection

**DOI:** 10.7554/eLife.14281

**Published:** 2016-02-03

**Authors:** Shengwei Hu, Jun Qiao, Qiang Fu, Chuangfu Chen, Wei Ni, Sai Wujiafu, Shiwei Ma, Hui Zhang, Jingliang Sheng, Pengyan Wang, Dawei Wang, Jiong Huang, Lijuan Cao, Hongsheng Ouyang

Hu S, Qiao J, Fu Q, Chen C, Ni W, Wujiafu S, Ma S, Zhang H, Sheng J, Wang P, Wang D, Huang J, Cao L, Ouyang H. 2015. Transgenic shRNA pigs reduce susceptibility to foot and mouth disease virus infection. *eLife* **4**:e06951. doi: 10.7554/eLife.06951.

Published June 19, 2015

In the published article, an earlier version of Supplementary file 1 was used by mistake, in which the sequences shown were not for shRNA construction.

The correct oligonucleotides for shRNA construction are now included in the corrected Supplementary file 1.

Supplementary file 1. Oligonucleotides for shRNA construction.

The article has been corrected accordingly.

