# Supplementary material for: Transgenic shRNA pigs reduce susceptibility to foot and mouth disease virus infection
Source: eLife. 2015 Jun 19;4:e06951. doi: 10.7554/eLife.06951 (PMC4502569; doi:10.7554/eLife.06951)
Supplement: Supplementary file 1. — Target sequences of shRNA used in this study. DOI: http://dx.doi.org/10.7554/eLife.06951.009 [file elife06951s001.docx]

**Supplementary file 1.** Target sequences of shRNA used in this study

| Target sites | Sequence（5 ’to 3’） |
| --- | --- |
| V1 | 5'-AA GCCACCTACTACTTCTCTGTTCAAGAGAGAAACAGCGCTTTGTCCTCTTTTTTTTACGCGT-3' |
| V2 | 5'-AA CTACGGTGGTGAGACACAATTCAAGAGAGAAACAGCGCTTTGTCCTCTTTTTTTTACGCGT-3' |
| V3 | 5'-AACAGCTTACCACAAGGAACCTTCAAGAGACTGCATCAGGTCCAACACATTTTTTTTACGCGT-3' |
| V4 | 5'-AA GAGAACTACGGTGGTGAGATTCAAGAGAGAAACAGCGCTTTGTCCTCTTTTTTTTACGCGT-3' |
| V5 | 5'-AA CCTTACACGGCTCCACACCTTCAAGAGAGAAACAGCGCTTTGTCCTCTTTTTTTTACGCGT-3' |
| V6 | 5'-AA CGGCCACCTACTACTTCTCTTCAAGAGAGAAACAGCGCTTTGTCCTCTTTTTTTTACGCGT-3' |
| V7 | 5'-AAGAGGACAAAGCGCTGTTTCTTCAAGAGAGAAACAGCGCTTTGTCCTCTTTTTTTTACGCGT-3' |
| V8 | 5'-AA AACTACGGTGGTGAGACACTTCAAGAGAGAAACAGCGCTTTGTCCTCTTTTTTTTACGCGT-3' |
| V9 | 5'-AA TACACGGCTCCACACCGTGTTCAAGAGAGAAACAGCGCTTTGTCCTCTTTTTTTTACGCGT-3' |
| V10 | 5'-AA ACGGTGGTGAGACACAAGTTTCAAGAGAGAAACAGCGCTTTGTCCTCTTTTTTTTACGCGT-3' |
| Scrambled control | 5'-ACGTAGCTAGCGTACGTACATTCAAGAGACACTTCCTCGAACATGATGTTTTTTTTACGCGT-3 |
