# Supplementary material for: Correction: Transgenic shRNA pigs reduce susceptibility to foot and mouth disease virus infection
Source: eLife. 2016 Feb 3;5:e14281. doi: 10.7554/eLife.14281 (PMC4755748; doi:10.7554/eLife.14281)
Supplement: Supplementary file 1. [file elife-14281-supp1.docx]

**Supplementary file 1.** Oligonucleotides for shRNA construction

| Target sites | Sequence |
| --- | --- |
| V1 | 5'-GCCACCTACTACTTCTCTGTTCAAGAGACAGAGAAGTAGTAGGTGGCTTTTTTGGAA-3’  5'-AATTTTCCAAAAAAGCCACCTACTACTTCTCTGTCTCTTGAACAGAGAAGTAGTAGGTGGCGTAC-3’ |
| V2 | 5'-CTACGGTGGTGAGACACAATTCAAGAGATTGTGTCTCACCACCGTAGTTTTTTGGAA-3’  5'-AATTTTCCAAAAAACTACGGTGGTGAGACACAATCTCTTGAATTGTGTCTCACCACCGTAGGTAC-3’ |
| V3 | 5'-GACTGAACTGCTTTACCGCTTCAAGAGAGCGGTAAAGCAGTTCAGTCTTTTTTGGAA-3’  5'-AATTTTCCAAAAAAGACTGAACTGCTTTACCGCTCTCTTGAAGCGGTAAAGCAGTTCAGTCGTAC-3’ |
| V4 | 5'-GAGAACTACGGTGGTGAGATTCAAGAGATCTCACCACCGTAGTTCTCTTTTTTGGAA-3’  5'-AATTTTCCAAAAAAGAGAACTACGGTGGTGAGATCTCTTGAATCTCACCACCGTAGTTCTCGTAC-3’ |
| V5 | 5'-CCTTACACGGCTCCACACCTTCAAGAGAGGTGTGGAGCCGTGTAAGGTTTTTTGGAA-3’  5'-AATTTTCCAAAAAACCTTACACGGCTCCACACCTCTCTTGAAGGTGTGGAGCCGTGTAAGGGTAC-3’ |
| V6 | 5'-CGGCCACCTACTACTTCTCTTCAAGAGAGAGAAGTAGTAGGTGGCCGTTTTTTGGAA-3’  5'-AATTTTCCAAAAAACGGCCACCTACTACTTCTCTCTCTTGAAGAGAAGTAGTAGGTGGCCGGTAC-3’ |
| V7 | 5'-GAGGACAAAGCGCTGTTTCTTCAAGAGAGAAACAGCGCTTTGTCCTCTTTTTTGGAA-3’  5'-AATTTTCCAAAAAAGAGGACAAAGCGCTGTTTCTCTCTTGAAGAAACAGCGCTTTGTCCTCGTAC-3’ |
| V8 | 5'-AACTACGGTGGTGAGACACTTCAAGAGAGTGTCTCACCACCGTAGTTTTTTTTGGAA-3’  5'-AATTTTCCAAAAAAAACTACGGTGGTGAGACACTCTCTTGAAGTGTCTCACCACCGTAGTTGTAC-3’ |
| V9 | 5'-TACACGGCTCCACACCGTGTTCAAGAGACACGGTGTGGAGCCGTGTATTTTTTGGAA-3’  5'-AATTTTCCAAAAAATACACGGCTCCACACCGTGTCTCTTGAACACGGTGTGGAGCCGTGTAGTAC-3’ |
| V10 | 5'-ACGGTGGTGAGACACAAGTTTCAAGAGAACTTGTGTCTCACCACCGTTTTTTTGGAA-3’  5'-AATTTTCCAAAAAAACGGTGGTGAGACACAAGTTCTCTTGAAACTTGTGTCTCACCACCGTGTAC-3’ |
| Scrambled control | 5'-ACGTAGCTAGCGTACGTACTTCAAGAGAGTACGTACGCTAGCTACGTTTTTTTGGAA-3’  5'-AATTTTCCAAAAAAACGTAGCTAGCGTACGTACTCTCTTGAAGTACGTACGCTAGCTACGTGTAC-3’ |

KpnI and EcoRI site overhang sequences were included for shRNA construction.
